# Bilateral Lung Transplantation for Congenital Pulmonary Arteriovenous Fistula with Intraoperative Venovenous ECMO Support: The First Case Report in China

**DOI:** 10.3389/fsurg.2022.861797

**Published:** 2022-05-31

**Authors:** Jialong Liang, Yuan Chen, Jintao Zhou, Mingfeng Zheng, Feng Liu, Shugao Ye, Jingyu Chen, Yong Ji

**Affiliations:** Department of Thoracic Surgery, The Affiliated Wuxi People’s Hospital of Nanjing Medical University, Wuxi, China

**Keywords:** bilateral lung transplantation, congenital pulmonary arteriovenous fistula, extracorporeal membrane oxygenation, hereditary hemorrhagic telangiectasia, case report

## Abstract

Pulmonary arteriovenous fistula (PAVF) is a rare pulmonary vascular lesion, more than 80% of which is caused by congenital abnormal development of pulmonary capillaries. The incidence of PAVF ranges from 2/100,000 to 3/100,000, with no difference in the male and female ratio. Congenital PAVF is often associated with hereditary hemorrhagic telangiectasia (HHT). In this article, we report a patient with only congenital PAVF that was successfully treated by bilateral lung transplantation (BLT) with intraoperative venovenous extracorporeal membrane oxygenation (ECMO) support because both lungs have been affected by PAVF and secondary pulmonary hypertension. To the best of our knowledge, this is the first report of BLT for PAVF in China and the second report that explains the clinical course of a patient to receive BLT for congenital PAVF without HHT. Some investigators have proposed lung transplantation as a definitive treatment, but the results are controversial. On the basis of the current condition of this patient, we believe lung transplantation is a viable option for certain patients, but the long-term effect remains to be studied.

## Introduction

Pulmonary arteriovenous fistula (PAVF) is a rare pulmonary vascular lesion, which means the pulmonary artery and its branches are interlinked with the corresponding vein (1). This results in pulmonary artery’s hypoxic blood being drained directly from the pulmonary vein to the left heart without oxygenation through pulmonary capillaries, creating a right-to-left shunt. In this article, we report a patient with only congenital PAVF that was successfully treated by bilateral lung transplantation (BLT).

## Case Report

Our patient is a 28-year-old woman. Since birth, she had cough and dyspnea, which significantly aggravated after physical activity and alleviated after resting. At that time, the presence of congenital heart disease was suspected, but all test results showed no pathological values, and therefore, no further investigation or treatment was performed. During the growth process, the symptoms did not improve. Six years ago, the patient felt the symptoms aggravated and was diagnosed with congenital PAVF after completing spiral computed tomography pulmonary angiography (CTPA). One month ago, she coughed up bright red blood suddenly, 50 ml at one time and 200 ml at another time, followed by respiratory distress and dyspnea. The lowest reach of blood oxygen saturation was 60%. Therefore, she received a pulmonary artery embolism in a local hospital. After the operation, the symptoms improved, but pulmonary effusion was diagnosed, and blood oxygen saturation had no significant improvement, so she was admitted to our hospital for lung transplantation on September 22, 2021.

**Figure 1:**
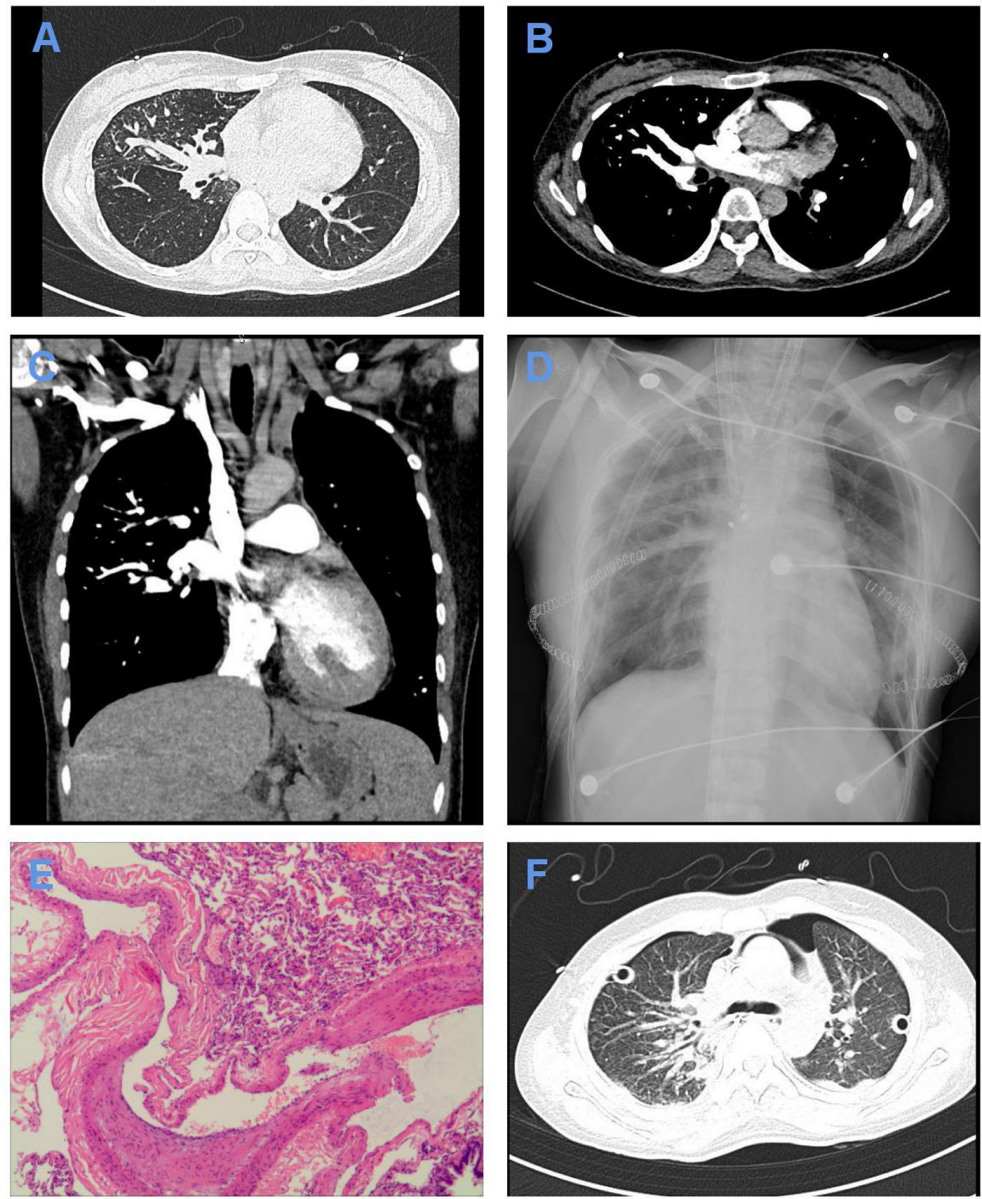
Imaging and pathological data of the patient. (**A**–**C**) Lung window (**A**), mediastinal window (**B**), and 3D window (**C**) of CTPA after admission showing simultaneous filling of the pulmonary artery and pulmonary vein in the right lung and the lower lobe of the left lung fill. (**D**) X-ray of the chest on the first postoperative day showing exudative changes in both lungs. (**E**) Pathological examination showing dilated blood vessels, different thicknesses of the blood vessel wall, partial anastomosis of the dynamic venous blood vessel wall, thickening of the intima of some blood vessels, and congestion and bleeding in the lung, conforming to the standard of PAVF with hemorrhage and secondary pulmonary hypertension. Scale: ×40. (**F**) Chest CT showing a small amount of pleural effusion on both sides, slight enlargement of the hilum of both lungs after double-lung transplantation, interstitial changes in both lungs with exudation, local pericardium thickening, and locally thickened pleura on both sides.

After admission, CTPA indicated simultaneous filling of the pulmonary artery and pulmonary vein in the right lung and the lower lobe of the left lung, scattered patchy fuzzy shadows in both lungs, especially in the upper lobe of the right lung, and high-density shadows in the posterior mediastinal area (Figure 1A–C). Lung function test resulted in the following: FVC=2.537(86.8%), FEV1=2.228(88.5%), and FEV1/FVC=87.8%.

PaCO_2_ was 31.6 mmHg and PaO_2_ was 38.2 mmHg at 20 L/min oxygen inhalation. The patient had respiratory failure, cyanosis of the lips, cyanosis of the extremities, and clubbing fingers (toes). The patient’s response to the medical treatment to relieve respiratory distress did not show any clinical improvement, and the condition progressively deteriorated. She was treated with high oxygen concentration and was unable to get out of bed.

Twenty-five days after admission, the patient received BLT instead of single-lung transplantation with intraoperative venovenous extracorporeal membrane oxygenation (ECMO) support under general anesthesia because both lungs have been affected by PAVF and secondary pulmonary hypertension. ECMO was placed in the right femoral vein and the right jugular vein. The patient received oxygen with an inspired oxygen fraction (FiO_2_) of 100%. Then, the patient was brought into the left lateral decubitus position. We entered the chest from the fifth rib. Intraoperative exploration showed massive intrathoracic adhesions, and tortuously hyperplastic vessels were seen on the surface of the right lung, chest wall, mediastinum, and hilum. We transplanted the right lung first and then the left lung. The cold ischemia time of the right lung was 6 h, and the cold ischemia time of the left lung was 8 h 30 min. Intraoperative blood loss was 1,600 mL, and blood transfusion was 1,450 mL. Postoperative circulation was stable, and oxygen saturation was unsatisfactory. Pathological examination showed the typical histological appearance of PAVF with hemorrhage and secondary pulmonary hypertension in both lungs (Figure 1E).

After surgery, she received assisted ventilation with a ventilator, anti-infection treatment with imipenem, immunosuppressive therapy with tacrolimus, expectorant treatment with ambroxol, and acid suppression therapy with omeprazole. The patient was also treated with norepinephrine ditartrate by an intravenous pump at a speed of 8 μg/min. Fourteen hours after surgery, the flow of ECMO was reduced to 1 L·min^−1^·m^−2^, and the air source was turned off. After 6 h, blood gas analysis showed that the ratio of arterial partial pressures of oxygen (PaO_2_) to FiO_2_ is 495 mmHg and the lactic acid level is 2.9 mmol/L. The patient was hemodynamically stable, so ECMO was removed. On the first postoperative day, an X-ray of the chest showed exudative changes in both lungs (Figure 1D). On the second postoperative day, the tubes were successfully removed and replaced with alternating high-flow oxygen inhalation and a noninvasive ventilator (BiPAP mode) after trials of spontaneous breathing. On the eighth postoperative day, tracheoscopy demonstrated a lack of roundness of bronchial anastomotic stoma, a small amount of yellowish-white sticky moss covering the left side, and more moss covering the right side. Multislice spiral CT of the chest showed a small amount of pleural effusion on both sides, slight enlargement of the hilum of both lungs after double-lung transplantation, interstitial changes in both lungs with exudation, local pericardium thickening, and locally thickened pleura on both sides (Figure 1F). Sputum culture results showed that drug-resistant *Klebsiella pneumoniae* was positive, so ceftazidime was added for anti-infection treatment. One month after the operation, the patient recovered well and was discharged successfully. She is now being followed up.

## Discussion and Conclusion

At present, ECMO has become a very good auxiliary tool in the perioperative period of lung transplantation. Intraoperative ECMO improves systemic perfusion during orthotopic lung transplantation and prevents ischemia-reperfusion injury. The use of ECMO has been shown to improve postoperative survival (2). VV ECMO drains pulmonary vein blood for oxygenation and decarboxylation and returns to the venous circulation. VV ECMO only improves oxygenation compared to VA ECMO (3), which is why we use VV ECMO. The most common catheter way is the femoral vein and jugular vein, with the tip of the femoral vein located in the inferior vena cava and the tip of the jugular vein located in the superior vena cava (4). This is exactly the way we use ECMO. More recently, a new approach has been to insert a single double-lumen tube through the jugular vein, but this approach may have limited flow (3). Another method is through bilateral femoral vein insertion. This method increases the risk of recirculation because the blood does not undergo systemic circulation well (3).

PAVF was first reported by Churton in 1897 as multiple pulmonary aneurysms. The disease was first confirmed by angiography by Smith in 1939. The incidence of PAVF ranges from 2/100,000 to 3/100,000, with no difference in the male and female ratio (5). More than 80% of the cases are caused by congenital abnormal development of pulmonary capillaries, and acquired diseases (such as cirrhosis), trauma, and surgery can also lead to PAVF. The clinical symptoms and severity of PAVF are closely related to the size of the lesion, mainly depending on the right-to-left shunt volume. When the shunt volume is larger than 20%, a series of manifestations of hypoxemia and heart failure can occur. The most common and early clinical symptoms, including dyspnea, hemoptysis, chest pain, and cough, occur in 8% of patients due to malformed blood vessel rupture, which can endanger life in severe cases. Typical PAVF triad refers to labored dyspnea, cyanosis, and clubbing fingers (toes). About 47%–80% of congenital PAVF also have hereditary hemorrhagic telangiectasia (HHT), and more than 20% of HHT patients develop PAVF. According to Curcao’s diagnostic criteria, a diagnosis of definite HHT is recognized if at least three of the following are present: spontaneous recurrent nosebleeds, mucocutaneous telangiectasia, visceral involvement, and an affected first-degree relative (6, 7). Therefore, our patient cannot be diagnosed with HHT but can be diagnosed with congenital PAVF.

Currently, there are three treatment methods for PAVF: surgery, transcatheter embolotherapy (TCE), and drug therapy. TCE is the preferred one. However, a study on 16 cases of diffuse PAVF showed that TCE did not obviously improve the profound hypoxemia. Some investigators have proposed lung transplant as a definitive treatment, but the results are controversial (8). So far, there are four cases of diffuse PAVF that have received lung transplantation successfully (9, 10, 11). Only one case of them did not complicate HHT (9). So, this is the second report that explains the clinical course of a patient to receive BLT for congenital PAVF without HHT, and this is our first time performing BLT for PAVF in China. On the basis of the current condition of this patient, we believe this is a viable option for certain patients, but the long-term effect remains to be investigated.

## Data Availability

The original contributions presented in the study are included in the article/supplementary material; further inquiries can be directed to the corresponding author/s.
